# Distribution of pathogens of nosocmial infections in a tertiary-level teaching hospital in Brazil

**DOI:** 10.1186/1753-6561-5-S6-P147

**Published:** 2011-06-29

**Authors:** EA Santos, F Siroma, DWCL Santos, RDF Feijó, N Cavalcante, CMM Pinto, R Richtmann, AMC Silva

**Affiliations:** 1 Hospitalar Infection Control Comission, Instituto de Infectologia Emilio Ribas, São Paulo, Brazil

## Introduction / objectives

The aim of this study was to describe the distribution of pathogens of nosocomial infections (NI) notified in a tertiary hospital of infectious diseases during 2010.

## Methods

From January to December 2010, all patients notified with NI according to CDC criteria definitions with agents isolated in cultures were enrolled.

## Results

In 2010 were identified 269 pathogens in 409 NI. The distribution including all sites of infection was: Coagulase Negative Staphylococcus (CNS) 20,6%, Pseudomonas aeruginosa 15,9%, Staphylococcus aureus 15,9%, Candida sp 7,8%, Escherichia coli 7,8%, Klebsiella sp 7,8%, Enterococcus sp 5,7%, Enterobacter sp 5,4%, Acinetobacter baumanii 3,4% and other agents 9,7%. The Staphylococcus sp sensibility to oxacilin were: 3,3% to CNS and S. aureus 29,7%; among P. aeruginosa, the sensibility to ceftazidima, cefepima, piperacilin-tazobactam, imipenem, meropenem and ciprofloxacin were, respectively, 64,4%, 62,2%, 59,5%, 72%, 55,5% and 69,5%. The production of ESBL among strains of E.coli and Klebsiella sp were 4,7% and 61,1%, respectively. Among the 175 nosocomial pneumonias notified, only 45 (26%) episodes had pathogens identified. The most important agents identified were P. aeruginosa, S. aureus, CNS and Enterobacter sp; among the bloodstream infection the most prevalent agents were CNS, S. aureus, Klebsiella sp and Candida sp and the most importants agents of urinary tract infections were E. coli, P. aeruginosa, Candida sp, Enterococcus sp and Klebsiella sp.

## Conclusion

The commonest agents in our institution were SCN and S. aureus, and P. aeruginosa among the gram negative bacteria. Differently to other tertiary hospitals in Brazil, we found a very low prevalence of Acinetobacter baumanii as agents of NI.

## Disclosure of interest

None declared.

